# Pre- and postnatal administration of *Lactobacillus reuteri* decreases TLR2 responses in infants

**DOI:** 10.1186/2045-7022-4-21

**Published:** 2014-06-25

**Authors:** Anna Forsberg, Thomas R Abrahamsson, Bengt Björkstén, Maria C Jenmalm

**Affiliations:** 1 Department of Clinical and Experimental Medicine, Unit of Autoimmunity and Immune Regulation, Division of Clinical Immunology, Linköping University, SE-581 85 Linköping, Sweden; 2 Department of Clinical and Experimental Medicine, Division of Pediatrics, Linköping University, Linköping, Sweden; 3 Institute of Environmental Medicine, Karolinska Institutet, Solna, Stockholm, Sweden; 4 School of Health and Medical Sciences, Örebro University, Örebro, Sweden

**Keywords:** *Lactobacillus reuteri*, Allergy, TLR, Cytokine, Chemokine, Infancy, Probiotics, Sensitization, Eczema

## Abstract

**Background:**

Mice models indicate that intact Toll like receptor (TLR) signaling may be essential for the allergy protective effects of diverse bacterial exposure observed in clinical trials and epidemiological studies. Probiotic supplementation with *Lactobacillus reuteri* from pregnancy week 36 and to the infant through the first year of life decreased the prevalence of IgE-associated eczema at two years (ClinicalTrials.gov NCT01285830). The effect of this supplementation on innate immune responses to bacterial products and the expression of associated TLRs were explored.

**Methods:**

Blood mononuclear cells were collected at birth, 6, 12 and 24 months from 61 infants and cultured with TLR2, 4 and 9 ligands. Cytokine and chemokine secretion was determined as well as TLR2, 4 and 9 mRNA expression.

**Results:**

Probiotic supplementation was associated with decreased LTA (lipoteichoic acid) induced CCL4, CXCL8, IL-1β and IL-6 responses at 12 months and decreased CCL4 and IL-1β secretion at 24 months. TLR2 mRNA expression was not affected by probiotic treatment.

**Conclusions:**

Decreased responses to TLR2, the main receptor for LTA from Gram positive bacteria, in probiotic treated children seem to be dependent on factors downstream of TLR mRNA expression.

## Introduction

Microbial exposure in early life educates the developing immune system, driving postnatal maturation of immune regulation [1]. The main receptors for bacterial products are the Toll like receptors (TLRs), which seem to be essential for the allergy protective effect of bacterial exposure during pregnancy [2].

Several studies have shown a lower incidence of eczema after probiotic supplementation pre- and postnatally, whereas prenatal or postnatal supplementation only have not generally been successful [3,4]. The underlying mechanisms of the allergy preventive effects are unclear, however. While probiotic supplementation may modulate adaptive immunity in infants [5-10], the effects on innate immune responses at the cellular level in infants are unknown. In one cohort showing that postnatal probiotic supplementation during the first six months of life had no clinical effect [11], no differences in innate immune responses were observed [12].

Previously, *Lactobacillus reuteri* supplementation in a randomized placebo controlled trial reduced IgE-associated eczema at the age of two years [13] but showed no protective effect on asthma development at the age of seven [14]. Infants supplemented with probiotics showed lower secretion of Th1/Th2 and anti-inflammatory cytokines and chemokines after allergen and mitogen stimulation as compared with infants receiving placebo [10]. These results suggest that *Lactobacillus reuteri* supplementation could enhance the immune regulatory capacity during infancy, possibly via innate-mediated mechanisms. This prompted us to investigate if the pre- and postnatal supplementation with *Lactobacillus reuteri* affects the innate cytokine and chemokine responses to bacterial products (the TLR2 ligand lipoteichoic acid (LTA) [15,16], the TLR4 ligand lipopolysaccharide (LPS) [17] and the TLR9 ligand CpG [18]), as well as expression of the associated receptors.

## Methods

### Study design

Sixty-one children, of whom 29 received probiotics and 32 placebo (Table 1), were selected based on availability of blood cell samples at multiple time points from a double-blind, randomized, placebo-controlled probiotic trial, in which 188 infants completed the study, ClinicalTrials.gov, NCT01285830 [13]. The infants included in this study had cells collected from at least three time points, *i.e.* birth, 6, 12 and 24 months. They were not statistically significantly different from the main trial with regards to the variables presented in Table 1. They all had a family history of allergic disease, *i.e.* at least one family member had eczema, asthma, gastrointestinal allergy, allergic urticaria, and/or allergic rhinoconjunctivitis. The pregnant women either received *Lactobacillus reuteri* ATCC 55730 or placebo daily from week 36 until delivery, and their infants continued from day 1–3 with the same product daily until 12 months of age. An informed consent was obtained from both parents before inclusion.

**Table 1:** Descriptive data of children included in the study

	***Lactobacillus reuteri % (n/N)***	**Placebo % (n/N)**	p-value*
Boys	55 (16/29)	50 (16/32)	0.82
First born	55 (16/29)	63 (20/32)	0.77
Caesarean delivery	14 (4/29)	3 (1/32)	0.16
Birth weight (g) (mean ± SE)	3656 ± 434	3469 ± 429	0.10*
Birth length (cm)	51.3 ± 2.0	50.6 ± 1.9	0.18*
Parental smoking	10 (3/29)	9 (3/32)	0.91
Furry pets	7 (2/29)	13 (4/32)	0.51
Maternal atopy	79 (23/29)	75 (24/32)	0.89
Paternal atopy	62 (18/29)	69 (22/32)	0.80
Breastfeeding			
3 months, exclusive	59 (17/29)	78 (23/32)	0.62
6 months, partial	83 (24/29)	78 (24/32)	0.80
Antibiotics			
0–12 months	34 (10/29)	16 (5/32)	0.18
12–24 months	48 (14/29)	53 (17/32)	0.83
Day-care			
0–12 months	0 (0/29)	3 (1/32)	0.35
12–24 months	86 (25/29)	94 (30/32)	0.82
Recurrent wheeze	3 (1/29)	6 (2/32)	0.63
Eczema	28 (8/29)	28 (9/32)	0.97
Allergic disease	34 (10/29)	38 (12/32)	0.87
Sensitisation	38 (11/29)	34 (11/32)	0.84
IgE-assoc. Disease	10 (3/29)	16 (5/32)	0.59
SPT (12 m cumulative)	10 (3/29)	16 (5/32)	0.37
SPT (24 m cumulative)	17 (5/29)	16 (5/32)	0.89
Egg	10 (3/29)	13 (4/32)	0.81
Milk	3 (1/29)	0 (0/32)	0.30
Cat	3 (1/29)	6 (2/32)	0.63
Birch	3 (1/29)	3 (1/32)	0.95
Spec IgE (kU/L)	34 (10/29)	31 (10/32)	0.85
Ovalbumin	7 (2/29)	22 (7/32)	0.15
b-lactoglobulin	24 (7/29)	9 (3/32)	0.19
fx5	31 (9/29)	28 (9/32)	0.85

*X*^*2*^ test. *t-test.

The *Lactobacillus* preparation consisted of freeze-dried *L. reuteri* suspended in coconut oil and peanut oil containing cryoprotective components, while the placebo preparation only comprised the vehicle. The daily intake corresponded to 1 × 10^8^ colony forming units (CFUs).

The children were monitored by research nurses and a final follow up was done by a pediatrician at 2 years of age, *i.e.* one year after the termination of treatment. Skin prick tests (SPTs) were done at 6, 12 and 24 months of age. Potentially allergic disease included eczema, recurrent wheeze, rhinoconjunctitivis, urticaria and gastrointestinal allergy. Eczema was classified as a pruritic, chronic, or chronically relapsing non-infectious dermatitis with typical features and distribution. Eczema was classified as IgE-associated if the infant was also sensitized, *i.e.* had at least one positive SPT and/or detectable circulating IgE antibodies to allergens. Wheeze was defined as an episode with obstructive airway symptoms, and recurrent wheeze was defined as three or more wheezing episodes, at least once verified by a physician. Allergy was defined as having at least one positive SPT and/or detectable circulating IgE (>0.35 kU/L), in addition to clinical symptoms consistent with eczema or recurrent wheeze. The eight children defined as allergic in this study all had IgE-associated eczema (n = 3 *L. reuteri*/n = 5 placebo). One child also had recurrent wheeze, one child allergic urticaria and one gastrointestinal allergy, while none of the children had allergic rhinoconjunctivitis. Non-sensitized children with symptoms (n = 8) and sensitized children without symptoms (n = 14) were not included in the analysis comparing allergic and non-allergic children. In the main trial, the prevalence of IgE-associated eczema at two years was lower in the *L reuteri* than the placebo group (8% vs. 21%, p = 0.02) [13].

### Ethics statement

This study was approved by the Regional Ethics Committee for Human Research at Linköping University (Dnr 99323) and registered at ClinicalTrials.gov (NCT01285830).

### Sample preparations

Cord blood was collected at birth and venous blood samples were drawn at 6, 12 and 24 months into heparinized vacutainers. Peripheral blood mononuclear cells (PBMC) were collected by Ficoll gradient centrifugation. Briefly, blood was layered on a Ficoll gradient, centrifuged and the PBMC layer was collected with subsequently washing and centrifugation steps. Cells were resuspended in freezing media consisting of 40% IMDM, 10% DMSO and 50% FCS. The cells were then placed in a freezing container at −70°C for 24 hours and thereafter stored in liquid nitrogen, pending analysis [19].

### Cell cultures stimulated with TLR ligands

PBMCs were thawed and stimulated with 1 ng/ml lipoteichoic acid (LTA) from *S. aureus* (InvivoGen, San Diego, USA), 1 ng/mL lipopolysaccharide (LPS) from *E. coli* K12 (Invivogen) or 1 μg/ml CpG ODN M362 (InvivoGen) in AIM V (Life Technologies AB, Täby, Sweden) with 20 μM mercaptoethanol (Sigma-Aldrich) at a concentration of 1 × 10^6^/ml and incubated for 24 hours at 37°C. Unfortunately, enough cells were not always available for all stimuli. Cell cultures were then centrifuged, supernatants were collected and cell pellets were lysed in RLT-buffer (Qiagen GmbH, Hilden, Germany) and stored at −70°C for later mRNA analysis. The mean viability of the freeze/thawed PBMCs was 85.2%.

### RNA extraction and quantitative real time PCR determination TLR2/4 and 9 mRNA and 18S rRNA

Total RNA was extracted from the control/spontaneous RLT-lysates using the RNeasy 96 plate-kit (Qiagen GmbH), according to the manufacturer’s instructions. Real-time PCR was conducted, as described previously [10]. Briefly, 1 μL of cDNA was mixed with 19 μL TaqMan Fast Universal Mastermix (Applied Biosystems, Paisley, UK) together with primers and probe for TLR2 mRNA (HS00152932_m1), TLR4 mRNA (HS01060206_m1), TLR9 mRNA (HS00370913_s1) (Applied Biosystems). The sequences for 18S rRNA primers and probe (Eurogentec S.A, Seraing, Belgium) have been described elsewhere [20].

### Measurement of cytokine and chemokine secretion by Luminex

The levels of pro-inflammatory cytokines and chemokines (IL-1β, IL-6, IL12p70, IFN-a, TNF, CCL4 and CXCL8) and the anti-inflammatory cytokine IL-10 in the cell supernatants were analysed with multiplex assay kits, according to the manufacturer’s instructions (Bio-Rad Laboratories, Hercules, CA, USA). The samples were analysed on a Luminex^100^ instrument (Biosource, Nivelles, Belgium) and the data was analysed with the software StarStation 2.0 (Applied Cytometry Systems, Sheffield, UK). The lower detection limit was 2,34 pg/mL for IL-1β, 33 pg/mL for IL-6, 1 pg/mL for IL-10, 0.5 pg/mL for IL12p70, 10 pg/mL for IFN-α, 20 pg/mL for CCL4 and CXCL8 and 3 pg/mL for TNF. Undetectable samples were given the value of half the cut off level. Comparisons of the TLR ligand induced cytokine and chemokine responses were made after the control value, *i.e.* responses from cells cultured in medium alone, was withdrawn. Detectable levels of IL-6, IL-10, CCL4, CXCL8 and TNF could be measured after all stimulations. After LPS and LTA stimulation, IL-1β levels could also be detected. Detectable IFN-α levels were observed only after stimulation with CpG.

### Statistics

As the data were not normally distributed, non-parametric tests were used. Comparisons between unpaired groups were analyzed with Mann–Whitney *U*-test, and correlations were analyzed with Spearman’s rank order correlation coefficient test. The student’s *t*-test and *χ*^2^-test was used to compare the background factors between the groups. Probiotic supplementation was included as dependent variable (placebo coded as 1, probiotic as 0) and IgE-associated allergic disease was included as independent variable in a logistic regression model, and the association between cytokine and chemokine secretion and these variables was investigated. P-values <0.05 were considered statistically significant. Calculations were performed with a SPSS statistical package version 19.0; SPSS Inc, Chicago, Ill. Undetectable samples were given the value of half the cutoff.

## Results

### Probiotic supplementation is associated with a reduced TLR2 ligand induced secretion of IL-1β, IL-6, CCL4 and CXCL8

**Figure 1:**
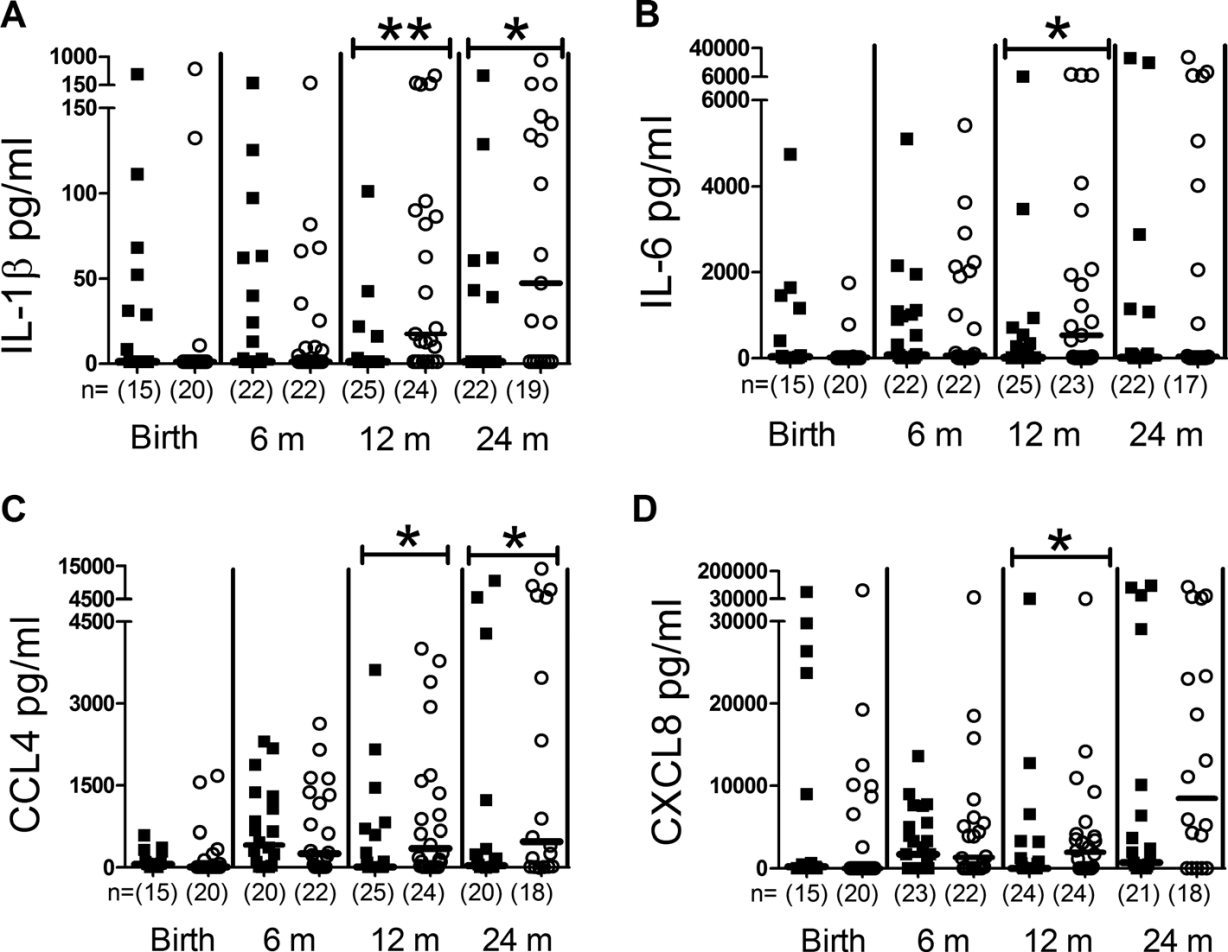
**LTA induced responses.** LTA induced IL-1β **(A)**, IL-6 **(B)**, CCL4 **(C)**, CXCL8 **(D)**, responses from peripheral blood mononuclear cells during the two first years in life in probiotic (filled squares) and placebo (open circles) treated children. Groups were compared using Mann–Whitney *U*-test, **p < 0.01, *p < 0.05. The number of analyzed samples is indicated (n), as well as the median values.

The levels of the chemokines CCL4 and CXCL8 were lower after stimulation with the TLR2 ligand LTA at 12 months in the probiotic than the placebo group (p = 0.014 and p = 0.043, respectively, Figure 1) and for CCL4 also at 24 months (p = 0.048, Figure 1). Furthermore, probiotic supplementation was associated with low levels of the pro-inflammatory cytokines IL-1β and IL-6 after LTA stimulation at 12 months (p = 0.002 and p = 0.017, respectively) and for IL-1β (p = 0.014) at 24 months. The cytokine and chemokine responses to LTA were statistically significantly lower at several time-points and showed similar trends at all ages in the probiotic compared with the placebo treated infants. Other measured cytokines showed no difference (Additional file 1: Table S1).

Although the probiotic treated infants showed the most clear and consistent differences in TLR2 responsiveness, probiotic supplementation was also associated with low CXCL8 responses to the TLR4 ligand LPS at birth (p = 0.040). No further differences were observed in responsiveness to TLR4 and TLR9 ligation (Additional file 1: Table S1).

### TLR mRNA expression in *Lactobacillus reuteri* and placebo supplemented infants

**Figure 2:**
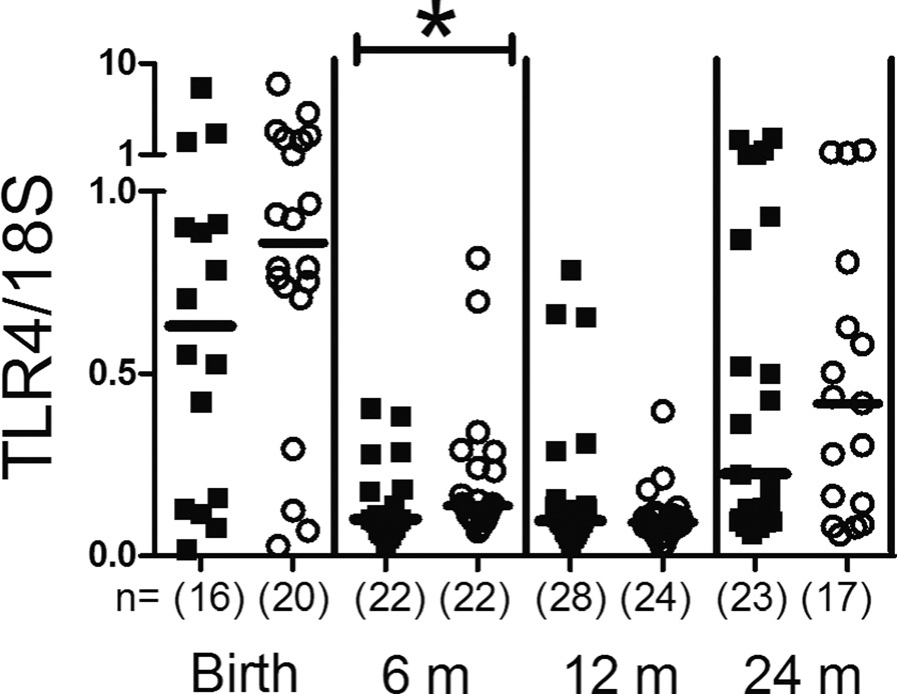
**TLR4 mRNA expression.** Comparison of TLR4 mRNA expression of peripheral blood mononuclear cells during the two first years in life in probiotic (filled squares) and placebo (open circles) treated children. The values represent TLR4/*18S* expression ratios. Groups were compared using Mann–Whitney *U*-test, *p < 0.05. The number of analysed samples is indicated (n), as well as the median values.

Since exposure to a farm environment has been suggested to up-regulate TLR mRNA expression [16-18], we investigated if probiotic supplementation affected the mRNA expression of TLR2/4 or 9. Neither the TLR2 nor the TLR9 expression differed between the two groups (Additional file 1: Table S1). The mRNA expression of TLR4 was significantly lower in the probiotic group than in the placebo group at 6 months of age, however (p = 0.039, Figure 2).

### TLR ligand induced cytokine and chemokine responses in allergic and non-allergic children

**Figure 3:**
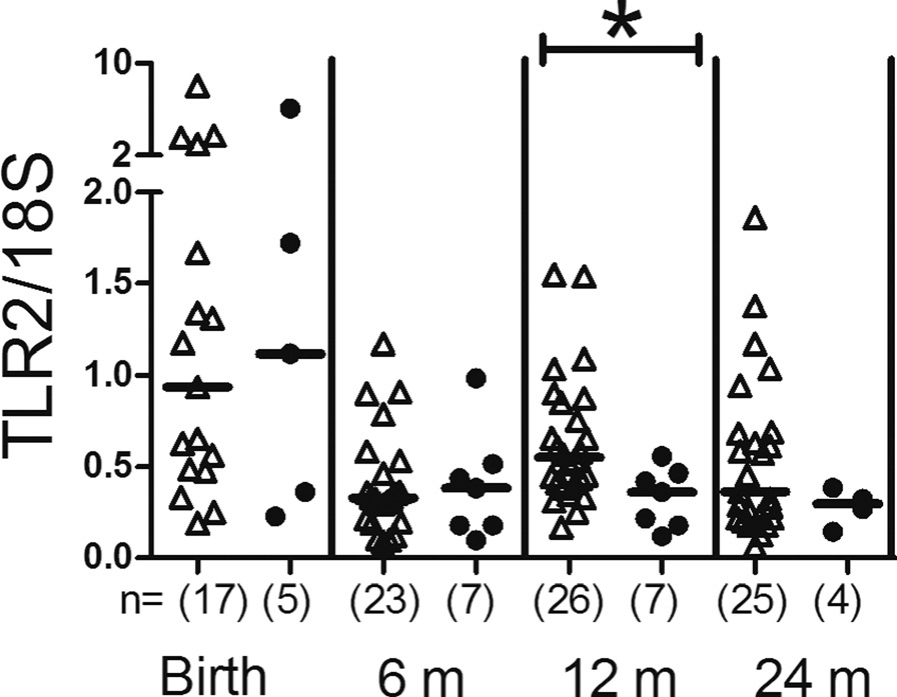
**TLR2 mRNA expression.** Comparison of TLR2 mRNA expression of peripheral blood mononuclear cells during the two first years in life in non-allergic (open triangles) and allergic children (filled circles). The values represent TLR2/*18S* expression ratios. Groups were compared using Mann–Whitney *U*-test, *p < 0.05. The number of analysed samples is indicated (n), as well as the median values.

No major differences in TLR ligand induced cytokine and chemokine responses were found between allergic (n = 3 *L. reuteri*/n = 5 placebo) and non-allergic children (n = 13 *L. reuteri*/n = 16 placebo) (Additional file 2: Table S2). Allergic infants secreted higher LPS-induced IL-1β levels at 6 months than non-allergic infants (p = 0.042) and lower CpG-induced TNF levels at 24 months (p = 0.048), however. TLR2 mRNA expression was higher at 12 months in non-allergic than allergic children (p = 0.021, Figure 3).

### TLR ligand induced cytokine and chemokine responses are associated with probiotic treatment independently of allergy development

Logistic regression analyses were performed to evaluate whether the relationship between cytokine responses was dependent on a parallel association with allergic disease. Probiotic supplementation and IgE-associated allergic disease were included in the model in order to control for potential confounding, whereas maternal or double atopic heredity were not, since these variables had no effect. Adjusting for allergy development had no major effect on the association between probiotic treatment and cytokine responses in this model (Additional file 3: Table S3).

### Correlation between TLR mRNA expression and TLR responsiveness

The TLR4 mRNA levels correlated with LPS induced cytokine and chemokine levels at the corresponding age in cord blood (CXCL8, p = 0.022, rho = 0.40) and at 24 months (TNF, CCL4, CXCL8, IL-1β, p < 0.001-0.04, rho = 0.35–0.66). At 6 months TLR9 mRNA expression correlated with CpG induced IL-10, IFNα, TNF, CCL4 (p < 0.001–0.01, rho = 0.40–0.51). TLR2 mRNA expression did only correlate at one time point with LTA induced cytokine and chemokine expression, (IL-10, 6 months, p = 0.011, rho = 0.34).

## Discussion

Pre- and postnatal supplementation with the Gram-positive probiotic bacterium *Lactobacillus reuteri* decreased TLR2 ligand induced proinflammatory cytokine and chemokine responses. The lower IL-1β, IL-6, CCL4 and CXCL8 responses to TLR2 stimulation in the probiotic treated infants were not dependent on differences in TLR2 mRNA expression in the probiotic and placebo group. Probiotic supplementation may be associated with an increased immune regulatory capacity during infancy, in line with our previous findings showing lower allergen responsiveness in the probiotic treated children [10].

Microbial stimulation is suggested for normal development of immune regulatory capacity [3,21]. The probiotic supplementation could then provide a source of TLR ligand exposure [3,21,22]. The lower responses to TLR2 stimulation could be dependent on an induction of regulatory macrophages responding to stimuli with lower secretion of pro-inflammatory cytokines and chemokines [23], as well as an induction of Tregs [24,25] which could be strain dependent [26]. Notably, induction of the anti-inflammatory cytokine IL-10 was not reduced by probiotic supplementation. Possibly, lower responses to TLR2 stimulation could be due to a phenomenon called lipoteichoic acid tolerance, which does not involve TLR2 expression but is dependent on a downstream effect involving IRAK [27]. Our data do indicate that the TLR2 mRNA expression and LTA induced cytokine and chemokine responses are not correlated.

Whether this down-regulation of TLR2 responses is also related to the decreased incidence of IgE-associated disease in probiotic treated children is unclear. Other studies suggest differences in TLR responsiveness between children who do and do not develop allergy [28-30]. While TLR ligand stimulation resulted in exaggerated responses at birth in children later developing allergy [29], these responses later seem to be attenuated in allergic compared to non-allergic children at the age of five years [28,30]. The number of allergic infants was probably too low to discriminate a significant and consistent difference in our study, although we did detect higher levels of TLR2 mRNA expression in non-allergic than allergic infants at one time point, 12 months of age. Anyway, the logistic regression analyses suggested that the effects of probiotic supplementation on TLR2 responsiveness were independent of allergy development in this study. Also, important to acknowledge was that there were no differences in antibiotic use and infections between probiotic and placebo treated infants which suggest that lower responses to TLR2 ligands is not detrimental in the protection against pathogens.

That probiotic supplementation may be associated with an increased immune regulatory capacity during infancy is also in line with studies suggesting that immune regulatory mechanisms are established at a later age in Sweden compared to Estonia [31], a country with higher microbial exposure and lower allergy prevalence than Sweden [32]. Estonian infants responded with lower levels of cytokines, both adaptive and innate, after allergen stimulation [31] and LPS stimulation, respectively, than Swedish infants. In concordance, neonatal antigen presenting cells are more quiescent in children born under traditional, *i.e.* Papua New Guinea, compared to modern environmental conditions, *i.e.* Australia [33]. This quiescent function could potentially be a protective mechanism learned *in utero* and associated with an enhanced immune regulatory capacity among infants living in conditions with a higher microbial burden.

## Conclusion

Decreased responses to TLR2, the main receptor for LTA from Gram positive bacteria, in probiotic treated children seem to be dependent on factors downstream of TLR mRNA expression.

## Competing interests

T Abrahamsson, M Jenmalm and B Björkstén have received honoraria from Biogaia AB for lectures.

## Authors’ contributions

AF, TA, BB and MJ conceived and designed the experiments. AF performed the experiments, AF, MJ and TA analysed the data. TA and MJ contributed reagents and materials. AF, MJ, BB and TA wrote the manuscript. TA was responsible for the clinical follow up of the children. All authors read and approved the final manuscript.

## Supplementary Material

Additional file 1: Table S1 TLR-ligand induced cytokine and chemokine (pg/ml) responses and TLR2 and −9 mRNA expression (TLR/18S ratio) in probiotic and placebo treated infants. Median, 1st and 4th quartile values are indicated. Click here for file

Additional file 2: Table S2 TLR-ligand induced cytokine and chemokine responses (pg/ml) in allergic and non-allergic infants. Median, 1st and 4th quartile values are indicated. Click here for file

Additional file 3: Table S3 Cytokine and chemokine responses after probiotic supplementation adjusted for IgE-associated allergic disease. Click here for file
