# Supplementary material for: Pre- and postnatal administration of Lactobacillus reuteri decreases TLR2 responses in infants
Source: Clin Transl Allergy. 2014 Jun 25;4:21. doi: 10.1186/2045-7022-4-21 (PMC4083862; doi:10.1186/2045-7022-4-21)
Supplement: Additional file 1: Table S1 — TLR-ligand induced cytokine and chemokine (pg/ml) responses and TLR2 and −9 mRNA expression (TLR/18S ratio) in probiotic and placebo treated infants. Median, 1st and 4th quartile values are indicated. [file 2045-7022-4-21-S1.docx]

Additional file 1: Table S1. TLR-ligand induced cytokine and chemokine (pg/ml) responses and TLR2 and -9 mRNA expression (TLR/18S ratio) in probiotic and placebo treated infants. Median, 1^st^ and 4^th^ quartile values are indicated.

|  |  | Cord Blood |  |  | 6 months |  |  | 12 months |  |  | 24 months |  |  |
| --- | --- | --- | --- | --- | --- | --- | --- | --- | --- | --- | --- | --- | --- |
|  |  | Probiotic | Placebo | p= | Probiotic | Placebo | p= | Probiotic | Placebo | p= | Probiotic | Placebo | p= |
| LPS | IL-1β | 51.0  (5.4-140.2) | 75.7  (19.2-165.7) | 0.5 | 38.2  (12.1-88.9) | 56.2  (1.17-119.4) | 0.7 | 73.2  (24.7-181.0) | 82.4  (43.0-198.0) | 0.5 | 89.0  (56.1-167.0) | 122.4  (87.5-441.4) | 0.1 |
|  | IL-6 | 2387  (1208-3675) | 4665  (1659-11715) | 0.072 | 677.0  (220.5-3077) | 2127  (392.7-8347) | 0.076 | 3136  (1537-5342) | 3035  (1857-6149) | 0.9 | 3921  (1189-8181) | 9707  (1267-19893) | 0.2 |
|  | IL-10 | 1.8  (1.2-8.4) | 10.4  (1.0-29.8) | 0.1 | 3.5  (1.0-11.3) | 13.2  (1.7-35.1) | 0.1 | 14.0  (2.8-40.1) | 18.4  (9.0-32.1) | 0.6 | 19.7  (7.2-45.2) | 56.4  (6.9-143.7) | 0.2 |
|  | TNF | 178.3  (75.2-358.9) | 446.8  (110.7-1278) | 0.1 | 260.9  (55.4-967.9) | 4057  (145.8-1354) | 0.3 | 707.0  (110.9-1342) | 974.8  (371.7-2062) | 0.3 | 1129  (538.9-3518) | 3161  (960.1-8098) | 0.053 |
|  | CCL4 | 1701  (772.6-3974) | 4332  (1278-7038) | 0.064 | 842.8  (247.0-3223) | 1607  (193.1-5232) | 0.3 | 1766  (514.7-3088) | 1622  (384.9-3877) | 0.9 | 3239  (1981-7967) | 8422  (2778-21082) | 0.066 |
|  | CXCL8 | 26087  (16604-50106) | 45829  (24647-179957) | **0.040** | 5930  (504.8-12466) | 10826  (10.0-39690) | 0.3 | 2562  (10.0-12240) | 2725  (10.0-23215) | 0.9 | 31693  (8224-57068) | 40674  (13333-107639) | 0.3 |
| LTA | IL-1β | 1.17  (1.17-52.2) | 1.17  (1.17-1.17) | 0.1 | 1.17  (1.17-45.5) | 1.17  (1.17-27.8) | 1.0 | 1.17  (1.17-1.17) | 15.7  (1.17-89.1) | **0.002** | 1.17  (1.17-40.2) | 47.3  (1.17-140.9) | **0.014** |
|  | IL-6 | 32.1  (16.4-1159) | 16.4  (16.4-16.4) | 0.050 | 76.7  (16.4-1034) | 64.7  (16.4-2058) | 0.8 | 16.4  (16.4-306.6) | 533.2  (16.4-2069) | **0.017** | 16.4  (16.4-343.8) | 46.1  (16.4-6240) | 0.3 |
|  | IL-10 | 0.5  (0.5-0.5) | 0.5  (0.5-0.5) | 0.6 | 0.5  (0.5-1.7) | 0.5  (0.5-3.4) | 0.4 | 0.5  (0.5-5.2) | 0.5  (0.5-9.8) | 0.6 | 0.5  (0.5-0.6) | 0.5  (0.5-34.2) | 0.2 |
|  | TNF | 9.2  (1.5-35.1) | 1.5  (1.5-13.4) | 0.4 | 1.5  (1.5-86.6) | 54.4  (1.5-233.1) | 0.068 | 7.3  (1.5-377.5) | 45.1  (1.5-327.7) | 0.9 | 17.3  (1.5-243.4) | 7.8  (1.5-922.9) | 0.8 |
|  | CCL4 | 64.7  (10.0-290.8) | 10.0  (10.0-220.9) | 0.4 | 411.9  (21.2-1240.0) | 255.6  (10.0-1338) | 0.5 | 10.0  (10.0-434.2) | 347.2  (15.0-1524) | **0.014** | 36.2  (10.0-317.5) | 476.7  (17.2-5295) | **0.048** |
|  | CXCL8 | 234.0  (10.0-23708) | 10.0  (10.0-9671) | 0.5 | 1716  (10.0-5524) | 1362  (10.0-5647) | 0.8 | 10.0  (10.0-1138.3) | 1957  (10.0-4086) | **0.043** | 716.9  (10.0-8234.4) | 8498  (10.0-26018) | 0.2 |
| CpG | IFN-α | 5.0  (5.0-5.0) | 5.0  (5.0-5.0) | 0.8 | 5.0  (5.0-19.2) | 5.0  (5.0-15.8) | 0.9 | 12.3  (5.0-20.2) | 5.0  (5.0-22.6) | 0.9 | 5.0  (5.0-25.5) | 5.0  (5.0-11.8) | 0.2 |
|  | IL-6 | 42.0  (16.4-237.0) | 286.0  (16.4-494.0) | 0.1 | 486.3  (124.5-1093) | 545.0  (88.5-967.0) | 0.7 | 190.4  (16.4-860.2) | 664.2  (16.4-1455) | 0.4 | 603.1  (161.7-1433) | 592.1  (152.4-1343) | 1.0 |
|  | IL-10 | 11.3  (3.4-21.4) | 16.5  (6.8-30.0) | 0.4 | 33.5  (13.1-72.6) | 18.8  (0.5-62.0) | 0.090 | 45.3  (7.3-92.2) | 37.7  (7.7-65.8) | 0.6 | 33.2  (14.0-51.6) | 26.7  (1.0-65.4) | 0.6 |
|  | TNF | 58.8  (22.1-81.4) | 52.8  (17.0-112.1) | 0.9 | 123.8  (13.5-305.4) | 105.2  (20.5-196.0) | 0.7 | 183.5  (42.7-559.0) | 152.6  (72.1-318.0) | 0.5 | 201.2  (42.5-316.9) | 91.5  (53.3-239.5) | 0.6 |
|  | CCL4 | 814.3  (63.9-1154) | 633.7  (10.0-2695) | 0.8 | 3059  (1623-5943) | 2615  (762.4-4670) | 0.3 | 1983  (1134-3155) | 30178  (1772-4644) | 0.1 | 2535  (1425-4073) | 2509  (1202-4788) | 0.8 |
|  | CXCL8 | 10.0  (10.0-8065) | 1042  (10.0-9750) | 0.9 | 10.0  (10.0-1680) | 10.0  (10.0-195.7) | 0.2 | 10.0  (10.0-3558) | 10.0  (10.0-532.6) | 1.0 | 10.0  (10.0-3046) | 760.5  (10.0-10125) | 0.2 |
| TLR2 |  | 0.8  (0.5-1.6) | 1.2  (0.5-3.0) | 0.4 | 0.3  (0.1-0.5) | 0.3  (0.2-0.5) | 0.3 | 0.4  (0.3-0.8) | 0.4  (0.3-0.7) | 0.4 | 0.3  (0.2-0.7) | 0.4  (0.2-0.6) | 1.0 |
| TLR9 |  | 12.8  (6.9-17.1) | 14.4  (11.5-19.2) | 0.3 | 21.0  (15.8-26.9) | 23.8  (19.1-37.9) | 0.1 | 16.9  (13.4-21.3) | 18.4  (13.6-24.8) | 0.6 | 16.2  (12.5-24.6) | 19.9  (14.6-24.1) | 0.2 |
