# Supplementary material for: Pre- and postnatal administration of Lactobacillus reuteri decreases TLR2 responses in infants
Source: Clin Transl Allergy. 2014 Jun 25;4:21. doi: 10.1186/2045-7022-4-21 (PMC4083862; doi:10.1186/2045-7022-4-21)
Supplement: Additional file 2: Table S2 — TLR-ligand induced cytokine and chemokine responses (pg/ml) in allergic and non-allergic infants. Median, 1st and 4th quartile values are indicated. [file 2045-7022-4-21-S2.docx]

Additional file 2: Table S2. TLR-ligand induced cytokine and chemokine responses (pg/ml) in allergic and non-allergic infants. Median, 1^st^ and 4^th^ quartile values are indicated.

|  |  | Cord Blood |  |  | 6 months |  |  | 12 months |  |  | 24 months |  |  |
| --- | --- | --- | --- | --- | --- | --- | --- | --- | --- | --- | --- | --- | --- |
|  |  | Non-allergic | Allergic | p= | Non-allergic | Allergic | p= | Non-allergic | Allergic | p= | Non-allergic | Allergic | p= |
| LPS | IL-1β | 51.0  (21.3-119.7) | 37.1  (12.8-1396) | 0.7 | 37.7  (1.17-68.3) | 99.9  (63.6-129.1) | **0.042** | 157.1  (41.3-284.3) | 62.3  (35.5-81.7) | 0.2 | 118.8  (56.4-294.2) | 96.2  (73.2-241.2) | 0.8 |
|  | IL-6 | 3207  (1501-4955) | 4138  (2146-40256) | 0.3 | 2127  (268.7-4434) | 1786  (375.7-10286) | 0.6 | 3246  (2580-6529) | 2438  (2114-5162) | 0.4 | 6804  (1393-14341) | 5064  (1834-9531) | 0.8 |
|  | IL-10 | 4.3  (1.0-9.1) | 16.2  (1.1-71.1) | 0.4 | 10.5  (3.3-20.3) | 13.7  (1.1-31.0) | 1.0 | 21.9  (12.0-41.8) | 10.2  (6.3-20.3) | 0.2 | 42.7  (7.4-151.8) | 20.1  (8.7-32.7) | 0.4 |
|  | TNF | 272.1  (121.1-790.0) | 742.2  (93.5-5352) | 0.4 | 398.4  (149.5-1285.0) | 691.5  (279.6-3227) | 0.5 | 900.0  (240.6-2222) | 443.3  (169.1-1016) | 0.3 | 1492  (690.6-6411) | 1875  (1053-3508) | 0.9 |
|  | CCL4 | 1863  (891.9-4244) | 7042  (646.4-14572) | 0.4 | 1454  (147.3-4101) | 4193  (701.9-6158) | 0.3 | 1699  (682.7-3749) | 1369  (458.0-2607) | 0.7 | 3734  (2013-12064) | 5787  (2424-6992) | 1.0 |
|  | CXCL8 | 40264  (22345-78617) | 28903  (23845-265761) | 0.9 | 9897  (10.0-23181) | 13877  (2088-41107) | 0.4 | 2713  (10.0-12639) | 5360  (435.0-14248) | 0.9 | 33455  (14139-100049) | 26901  (6881-57405) | 0.5 |
| LTA | IL-1β | 1.17  (1.17-19.7) | 1.17  (1.17-56.2) | 0.8 | 1.17  (1.17-13.6) | 1.17  (1.17-18.6) | 0.7 | 2.15  (1.17-87.3) | 1.17  (1.17-17.4) | 0.6 | 1.17  (1.17-64.1) | 1.17  (1.17-139.6) | 0.9 |
|  | IL-6 | 16.4  (16.4-234.0) | 16.4  (16.4-829.2) | 0.8 | 76.7  (16.4-2064) | 39.7  (27.6-1374) | 1.0 | 16.4  (16.4-3775) | 709.7  (26.7-1716) | 0.6 | 25.5  (16.4-1375) | 45.1  (16.4-9053) | 0.9 |
|  | IL-10 | 0.5  (0.5-0.5) | 0.5  (0.5-1.3) | 0.8 | 0.5  (0.5-1.4) | 0.5  (0.5-3.1) | 0.4 | 0.5  (0.5-12.4) | 0.5  (0.5-2.5) | 1.0 | 0.5  (0.5-5.8) | 0.5  (0.5-37.8) | 0.6 |
|  | TNF | 1.5  (1.5-26.3) | 1.5  (1.5-20.4) | 0.8 | 5.4  (1.5-87.1) | 83.6  (1.5-201.7) | 0.4 | 1.5  (1.5-387.5) | 70.7  (1.5-159.7) | 0.8 | 7.1  (1.5-219.6) | 14.3  (3.8-2366) | 0.5 |
|  | CCL4 | 10.0  (10.0-307.4) | 10.0  (10.0-69.3) | 0.6 | 381.3  (43.9-1437) | 212.5  (10.0-732.0) | 0.3 | 10.0  (10.0-1029.7) | 273.5  (10.0-710.5) | 0.7 | 172.5  (10.0-1060) | 13.0  (10.0-6501) | 0.6 |
|  | CXCL8 | 10.0  (10.0-15885) | 10.0  (10.0-39639) | 0.9 | 3932  (10.0-7749) | 1587  (10.0-5209) | 0.6 | 47.5  (10.0-3328) | 557.0  (10.0-2602) | 0.9 | 4145  (501.2-19755) | 217.2  (10.0) | 0.7 |
| CpG | IFN-α | 5.0  (5.0-5.0) | 5.0  (5.0-8.0) | 0.5 | 5.0  (5.0-18.6) | 7.8  (5.0-17.8) | 1.0 | 14.1  (5.0-23.9) | 5.0  (5.0-23.2) | 0.4 | 5.0  (5.0-16.2) | 12.6  (6.5-19.1) | 0.4 |
|  | IL-6 | 222.5  (16.4-452.2) | 30.4  (16.4-450.9) | 0.6 | 576.7  (52.5-1374) | 461.6  (257.3-1025) | 0.9 | 135.3  (16.4-670.0) | 1126  (282.9-1583) | 0.1 | 603.1  (250.9-1687.7) | 518.2  (56.7-1432) | 0.5 |
|  | IL-10 | 16.5  (6.3-19.6) | 10.6  (3.4-36.6) | 0.8 | 39.2  (7.7-76.6) | 37.5  (0.5-51.1) | 0.3 | 53.8  (14.7-71.1) | 22.7  (0.5-66.9) | 0.3 | 35.0  (14.8-77.4) | 1.5  (0.5-37.7) | 0.065 |
|  | TNF | 58.8  (15.1-102.8) | 22.1  (8.2-93.2) | 0.6 | 155.0  (21.9-283.8) | 142.2  (58.1-275.3) | 0.9 | 207.9  (88.5-472.9) | 152.6  (18.5-302.7) | 0.4 | 175.4  (61.3-276.4) | 26.4  (1.5-154.2) | **0.048** |
|  | CCL4 | 844.7  (10.0-1872) | 295.0  (5.0-2306) | 0.6 | 2553  (707.6-5705) | 3522  (1383-4304) | 1.0 | 2170  (1088-3030) | 2281  (521.7-4074) | 0.9 | 3701  (1202-5487) | 2756  (436.0-6489) | 0.7 |
|  | CXCL8 | 1196  (10.0-18825) | 2619  (10.0-5429) | 0.9 | 10.0  (10.0-10.0) | 615.2  (10.0-3006) | 0.5 | 10.0  (10.0-10.0) | 10.0  (10.0-3466) | 0.7 | 10.0  (10.0-8370) | 10.0  (10.0-3178) | 0.5 |
