# Supplementary material for: Pre- and postnatal administration of Lactobacillus reuteri decreases TLR2 responses in infants
Source: Clin Transl Allergy. 2014 Jun 25;4:21. doi: 10.1186/2045-7022-4-21 (PMC4083862; doi:10.1186/2045-7022-4-21)
Supplement: Additional file 3: Table S3 — Cytokine and chemokine responses after probiotic supplementation adjusted for IgE-associated allergic disease. [file 2045-7022-4-21-S3.docx]

**Additional file 3: Table S3**. Cytokine and chemokine responses after probiotic supplementation adjusted for IgE-associated allergic disease

| Placebo | | | |  | Adjusted for allergy | | | |
| --- | --- | --- | --- | --- | --- | --- | --- | --- |
| Age | **Stimuli** | | **p** | **OR** | **(95% CI)** | **p** | **OR** | **(95% CI)** |
| Cord blood | LPS | CCL4 | 0.02 | 6.4 | 1.44-28.5 | 0.2 | 3.0 | 0.476-18.5 |
|  | LTA | IL-6 | 0.04 | 0.21 | 0.046-0.912 | 0.2 | 0.34 | 0.053-2.09 |
|  |  | IL-1β | 0.05 | 0.20 | 0.041-0.991 | 0.1 | 0.21 | 0.028-1.61 |
| 12 months | LTA | IL-1β | 0.002 | 8.0 | 2.12-29.2 | 0.004 | 17.2 | 2.54-116 |
|  |  | IL-6 | 0.04 | 3.5 | 1.10-11.5 | 0.05 | 5.7 | 1.01-32.1 |
| 24 months | LPS | CCL4 | 0.06 | 3.5 | 0.945-13.0 | 0.02 | 9.2 | 1.37-61.5 |
|  | LTA | IL-1β | 0.02 | 6.4 | 1.17-15.4 | 0.008 | 23.4 | 2.28-239 |
|  |  | CCL4 | 0.07 | 3.6 | 0.924-13.8 | 0.01 | 23.6 | 2.11-263 |
|  |  | IL-8 | 0.09 | 6.5 | 1.60-26.4 | 0.003 | 45.6 | 3.67-591 |
